# Impact of COVID-19 on routine immunization in Oyo State, Nigeria: trend analysis of immunization data in the pre- and post-index case period; 2019-2020

**DOI:** 10.11604/pamj.2022.41.54.28575

**Published:** 2022-01-20

**Authors:** Olaniyan Akintunde Babatunde, Muideen Babatunde Olatunji, Olugbade Robertson Omotajo, Olukorede Ifedolapo Ikwunne, Adekunbi Mofoyeke Babatunde, Eunice Titilayo Nihinlola, Grace Funmilayo Patrick, David Magbagbeola Dairo

**Affiliations:** 1 Oyo State Primary Health Care Board, State Secretariat, Agodi, Ibadan, Oyo State, Nigeria,; 2 Oriire Local Government Health Authority, Ikoyi-Ile, Oyo State, Nigeria,; 3 Lagos State Internal Revenue Service, Alausa, Ikeja, Lagos State, Nigeria,; 4 Department of Epidemiology and Medical Statistics, Faculty of Public Health, College of Medicine, University of Ibadan, Oyo State, Nigeria

**Keywords:** COVID-19, routine immunization, index case, Nigeria

## Abstract

**Introduction:**

the response to COVID-19 pandemic has posed new obstacles to the fragile health system, most especially in the area of vaccination across much of Africa. As the response to the pandemic intensifies through the application of non-pharmacologic interventions as well as enforcement of the lockdowns across African cities, there is a significant risk that more children will miss out on life-saving vaccines that can prevent childhood killer diseases. This study was therefore conducted to look at the impact of the COVID-19 pandemic on routine immunization in Oyo State, Nigeria.

**Methods:**

we conducted a descriptive secondary analysis of immunization data between July 2019 and August 2020. These data were retrieved from the monitoring and evaluation unit of Oyo State Primary Health Care Board. The data were extracted from the original paper format and entered into Excel sheets. Line graphs were plotted to compare the trends of the coverage rates before and after the index case of the COVID-19 pandemic.

**Results:**

the average coverage rates for Bacillus Calmette-Guérin (BCG) before and after index case were 85.8% and 82.1% respectively, while it was 63.5% and 60.0% for HBV0. For the co-administered vaccines at 14 weeks, Penta 3, OPV 3, PCV 3 and IPV coverage rates dropped from 76.1%, 75.4%, 75.1% and 73.5% to 72.0%, 71.4%, 72.0% and 71.9% respectively. The average coverage rates for yellow fever and measles dropped sharply from 77.0% and 74.5% and 64.6% and 58.6% respectively. The average drop-out rates for the pre-and post-index case periods were 5.0% and 4.7% respectively. For the planned fixed and outreach sessions, none of the monthly sessions met the target of 100.0% in the post-index case period.

**Conclusion:**

decreased vaccination coverage for vaccine-preventable diseases could cause parallel outbreaks with COVID-19 and further exacerbate the strain on health systems attempting to end the acute phase of this pandemic. Therefore, as the dramatic second wave unfolds, the Government of Nigeria must take deliberate steps to strike a balance between a fresh lockdown and the imperative of uninterrupted social service. In this wise, it must remain committed to a timely vaccination program.

## Introduction

The recent certification of the African region as a polio-free continent has been applauded by many people across the globe, most especially public health experts who labored so much to achieve the feat [1,2]. It is on record that huge amount of resources was deployed to make the success possible and as a result, the imperative of sustaining the resultant gain cannot be over-emphasized. A key strategy in the attainment of a polio-free Africa was routine immunization which made and still makes vaccines available for children under 23 months based on national protocols [3]. However, since the outbreak of COVID-19, this cardinal child survival strategy has come under a huge threat across most parts of the developing world [4]. Starting in the Chinese industrial city of Wuhan in 2019, COVID-19 pandemic has spread rather quickly to encircle the whole globe [5]. Currently, over 100 million people have been affected worldwide with more than 2 million deaths [6]. The advent of the pandemic has also meant different things for different health care consumer groups. While, at the higher level of clinical care, it has revealed the widespread vulnerabilities of the public health system in many significant ways, on the other hand, the lower level of the national service stratum has tended to suffer largely from the effects of non-pharmacologic preventive measures put in place in many countries to limit the spread of the pandemic [7]. Prolonged lockdowns in these countries have affected access to essential health care services with many children kept away from the monthly routine immunization schedules. Globally, an estimated 13.5 million children missed out of routine immunization in 2020 expectedly due to diversion of emphasis to the control of the deadly pandemic [8]. All public investment resources; human, material and financial were concentrated on fighting the scourge with most other services suffering atrociously. For the low-end of the service scale, situation was further compounded by the historic supply chain disruption which led to the scarcity of vaccines and other essential medicines in many parts of low-and middle-income countries [9]. In consequence, mass immunization campaigns were either discontinued or suspended for a period of time with the Global Polio Eradication Initiative (GPEI) leading the clamor for the suspension of outbreak response campaign till June 1^st^, 2020 [10].

A review of administrative routine immunization data between 2018 and June 2020 from 15 African countries showed that 13 of the countries experienced a decline in the monthly average number of vaccine doses provided. Six had more than 10% decline [11]. In Nigeria, thematic-based assessment of routine immunization performance during the first wave of the pandemic revealed a sharp drop of 15% in service delivery [12]. This was in contrast to the healthy achievements of the past years. For example, according to the 2018 Nigeria Demographic and Health Survey, vaccination coverage in Nigeria has shown an upward shift over the past 10 years. The percentage of children aged 12-23 months who received all basic vaccinations increased from 23% in 2008 to 31% in 2018 [13]. The percentage of children who received none of the basic vaccinations declined from 29% to 19% during the same period [13]. While these trends were no doubt commendable, nonetheless, they still fell far short of the 90% coverage targeted under the sustainable development goal 3. In Oyo State, there is a long standing history of poor performance in routine immunization with coverage oscillating between 22% and 33% in the pre-COVID-19 era [13]. Right now, the realization that SARS COV-2 virus will be around for much longer time than previously expected has now necessitated a re-balancing of the global health agenda in such a way that stronger focus is being mounted on other paramount health issues especially immunization to prevent total collapse. Refocusing in this context implies adequate health system resourcing for better integrated care and management of patients across disease spectrum. Though, health system strengthening is required at all levels of care, this is however most crucial at the primary health care level in Nigeria which provides services to more than 70% of the populace, especially in the rural areas [14]. Primary health care (PHC) also represents the platform through which the welfarist policies of government are delivered to children and pregnant women in the country. This study was therefore conducted to look at the impact of COVID-19 pandemic on routine immunization along two abbreviated time frames: before and after the index case.

**Objectives:** i) to determine the coverage rates of all the antigens used for the routine immunization. ii) to assess the planned fixed and outreach immunization sessions during the study period. iii) to calculate the pentavalent vaccine drop-out rate during the study period. iv) to assess the impacts of COVID-19 pandemic on the routine immunization during the study period.

## Methods

**Study area:** Oyo State is located in the South-West geopolitical zone of Nigeria. It is in the tropical rainforest. The mean monthly temperature is in the range of 25°C to 31°C. The mean annual temperature ranges from 68°F to 93°F for the entire state. The hottest and coldest months of the year are February and December respectively. The average annual percentage of humidity is 81%. July is the most humid while February is the least humid. The state has a projected 2019 population of 8, 635, 793 using an annual growth rate of 3.4% and 2006 population figure as the baseline [15]. The populations of under-one and under-five years were 345,432 and 1,727,159 respectively. In 2019, three supplemental immunization activities (SIAs) were conducted while two SIAs were carried out in 2020. The recently conducted SIAs for yellow fever and meningitis were done in November 2020 with the target age for each between 9 months to 44 years.

**Study setting:** the State has a total of 733 health facilities offering routine immunization services distributed across 33 local government areas (LGAs). There are three types of health facilities in the LGAs; primary health care centre, primary health clinic and health post. There are primary health care centres (1 per ward) in each of the LGAs, while each ward has an average of 2 to 5 primary health clinics and health posts. The health facilities were populated by different cadres of the workforce; primary health care coordinators (these consist of Medical Officers of Health or in their absence, the most senior health workers), nurses/midwives, community health officers (CHOs), community health extension workers (CHEWs), medical laboratory scientists, medical record officers, pharmacy technicians, health assistants and attendants. Activities carried out in these health facilities include; antenatal clinic, infant welfare clinic, immunization services, nutritional services, laboratory services, health education, treatment of common diseases, etc. Routine immunization (RI) services are offered at least once a week in all the health facilities and are being managed by the routine immunization (RI) focal persons. There is a 24-hour daily operation in a few of the health facilities while the majority do not have human capacity for such operation. The coverage rate of the facilities depends on so many factors and those facilities located in the urban areas had higher patronage than their rural counterparts. Information on routine immunization flows from both the public and private health facilities to the LGA immunization officer who collates the data and forwards same to the LGA monitoring and evaluation officer. Aggregated data are uploaded into the DHIS2 from where it will be accessed by the Federal Ministry of Health.

**Study design:** we carried out a secondary analysis of immunization data for Oyo State for the period of July 2019 to August 2020.

**Data source:** data on vaccine-preventable diseases from all the 33 LGAs of Oyo State from July 2019 to August 2020 were obtained from the monitoring and evaluation unit of Oyo State Crimary health care Board (OYPHCB).

**Vaccination schedule:** the routine immunization schedule in Nigeria involves 5 visits to prevent against the vaccine-preventable diseases (VPDs) which include Tuberculosis, Polio, Diphtheria, Tetanus, Pertussis, Haemophilus influenza, Hepatitis B, Pneumococcal diseases, Yellow fever and Measles [16]. During the visits, the following vaccines are used for vaccination and the cumulative data of which were analyzed for the abridged study period: bacille calmette-Guérin (BCG) vaccine, Hepatitis B vaccine (HBV), bivalent Oral Polio vaccine (bOPV), Pentavalent vaccine (Penta), Pneumococcal conjugate vaccine (PCV), inactivated polio vaccine (IPV), Measles vaccine, Yellow fever vaccine (YF), Tetanus vaccine [16].

**COVID-19 lockdown in Nigeria:** in order to stem the tide of the rising cases of COVID-19, the Government of Nigeria (GoN) announced a lockdown starting at midnight in March 30, 2020 and ended in May 4^th^, 2020 [17]. It was a total ban on movement, social and economic activities in three major cities (Lagos, Ogun and Abuja) worst hit by the novel coronavirus [18]. Thereafter, other cities with rising cases of the virus were equally locked down. Providers with essential services were exempted and these include health, law enforcement, utility and telecommunications [17]. For the public, urgent medical care and limited access to food items were allowed. Health system was affected, most especially at the primary health care level where immunization activities were grossly disrupted. Due to the lockdown, outreach activities were suspended, vaccine supply chains both at national and state levels were interrupted. The public patronage for the routine immunization was at the lowest ebb. Monitoring and supervision of immunization sessions both at the fixed posts and outreach centers were upended due to the fear of COVID-19 by the health workers.

**Data management:** data were sorted; cleaned and relevant variables extracted using Microsoft Excel 2010. The following variables were used in the final analysis: HBV0, Penta, IPV3, OPV 3, Yellow Fever, Measles, Penta drop-out rate, fixed sessions, outreach sessions and date of analysis (months). Descriptive statistics including line graphs were plotted to compare the trends of the coverage rates before and after the index case of COVID-19 pandemic.

**Ethical consideration:** ethical approval for the study was obtained from the Oyo State Ministry of Health. We maintained the confidentiality of subjects by excluding all identifying information such as name and address from the analysis. Data were put in a pass-worded file and stored in a computer accessible only to the principal investigator.

## Results

**Figure 1:**
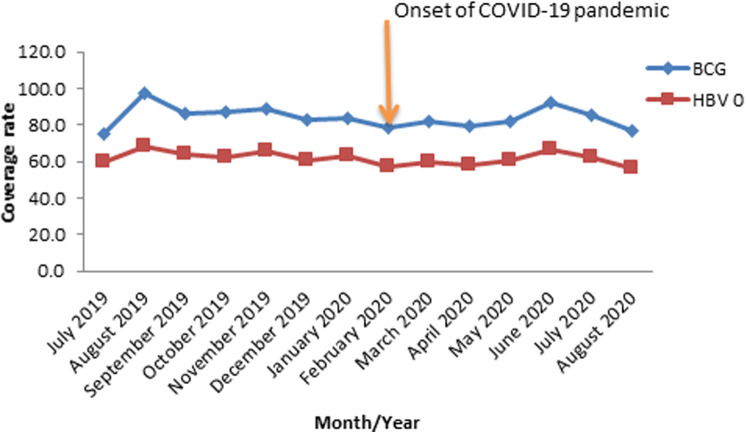
routine immunization coverage rates for BCG and HBV0 in Oyo State, South-Western Nigeria; 2019 - 2020

**Routine immunization coverage rate for BCG and HBV0 in Oyo State, South-Western Nigeria; 2019-2020:** the average coverage rates for BCG before and after COVID-19 index case were 85.8% and 82.1% respectively. The coverage rate for BCG peaked in August 2019 (97.0%) and was lowest (77.1%) in August 2020 during the course of the outbreak. The average coverage rates for HBV0 before and after COVID-19 outbreak were 63.5% and 60.0% respectively. The highest (75.3%) coverage rate for HBV0 was recorded in August 2019 while the lowest coverage rate (56.1%) was documented in August 2020 after the outbreak of COVID-19 in February 2020 (Figure 1).

**Figure 2:**
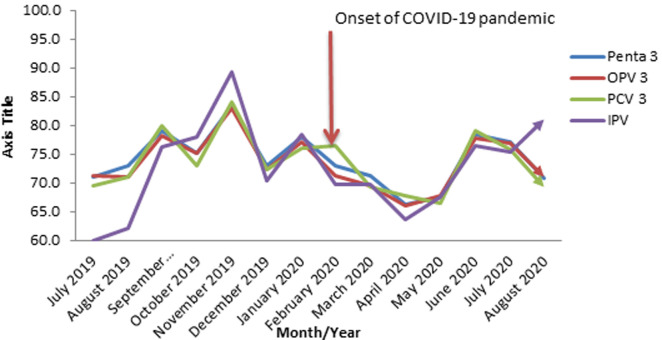
routine immunization coverage rates for Penta 3, OPV3, PCV 3 and IPV in Oyo State, South-Western Nigeria; 2019 - 2020

**Routine immunization coverage rates for Penta 3, OPV3, PCV 3 and IPV in Oyo State, South-Western Nigeria; 2019-2020:** for the co-administered vaccines, a sinusoidal wavelike pattern that peaked in November 2019 was recorded before the outbreak of COVID-19 while a rapid decline occurred from February to April 2020 before it started to rise again and then peaked around June 2020. The average coverage rates for Penta 3, OPV3, PCV3 and IPV before COVID-19 outbreak were 76.1%, 75.4%, 75.1% and 73.5% respectively, while the coverage rates after the index case were 72.0%, 71.4%, 72.0% and 71.9% respectively. The majority of these co-administered antigens had the lowest coverage rates in April 2020, two months after the onset of the pandemic (Figure 2).

**Figure 3:**
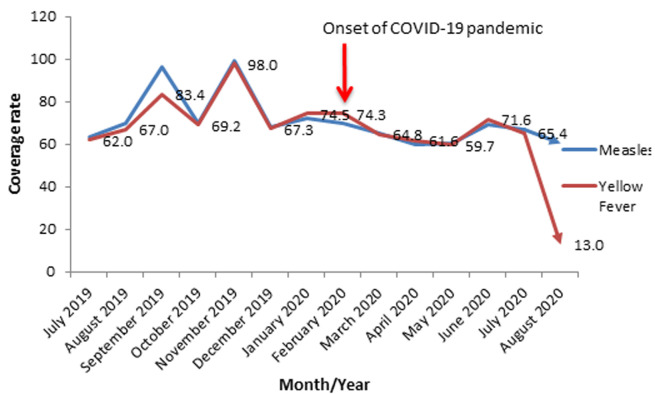
routine immunization coverage rates for measles and yellow fever in Oyo State, South-Western Nigeria; 2019-2020

**Routine immunization coverage rates for measles and yellow fever in Oyo State, South-Western Nigeria; 2019-2020:** a wavelike pattern was also noticed before the onset of COVID-19 while a gradual decrease occurred from February to May 2020 followed by a slight increase from May to June 2020. A sharp decline occurred from July to August 2020 (Yellow fever). The average coverage rates for measles before and after COVID-19 outbreak were 77.0% and 64.6% respectively. The average coverage rates for Yellow fever before and after COVID-19 outbreak were 74.5% and 58.6% respectively. However, a large discrepancy was noticed between measles and yellow fever coverages in August 2020 (Figure 3).

**Figure 4:**
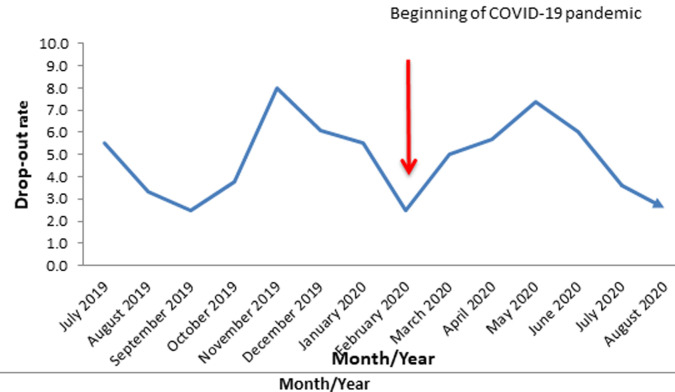
penta drop-out rate in Oyo State, Southwestern Nigeria; 2019 - 2020

**Penta drop-out rate in Oyo State, South-Western Nigeria; 2019-2020:** the highest drop-out rate was witnessed in November 2019 (8.0%) and started declining until February 2020 (the start of the outbreak) when it started rising again and got to a peak in May 2020 (7.4%) and thereafter descending. The average drop-out rates for the pre-and post-index case periods were 5.0% and 4.7% respectively (Figure 4).

**Figure 5:**
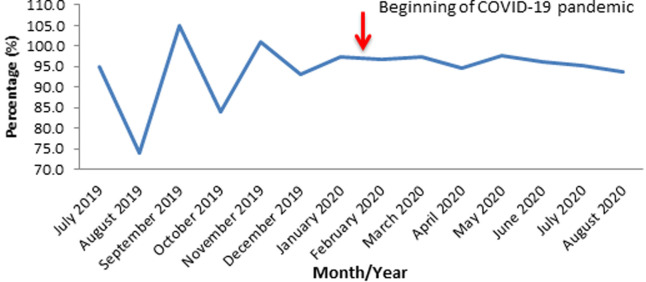
routine immunization planned fixed sessions conducted in Oyo State, South-western Nigeria; 2019 - 2020

**Planned fixed routine immunization sessions conducted in Oyo State, South-Western Nigeria; 2019-2020:** in the pre-index case period, more sessions were conducted than what was planned for in September 2019 (105.0%) and November 2019 (101.0%), while none of the monthly sessions met 100.0% target in the post-index case period. However, the least planned fixed-session was recorded in August 2019. The average planned fixed sessions recorded for pre-and post-index case periods were 92.8% and 95.9% respectively (Figure 5).

**Figure 6:**
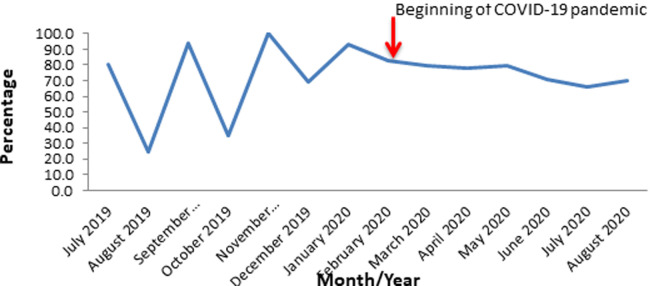
routine immunization planned outreach sessions conducted in Oyo State, South-western Nigeria; 2019 - 2020

**Planned outreach routine immunization sessions conducted in Oyo State, South-Western Nigeria; 2019-2020:** throughout the period under the review, it was only in November 2019 that 100.0% target was achieved. However, the lowest proportion of the sessions conducted was also reported in the pre-index-case period of August 2019 (25.0%). Whereas in the post-index case period, the least proportion of sessions conducted was in the month of July 2020 (66.0%). The average proportions of the outreach sessions conducted for pre- and post-index case periods were 71.0% and 75.2% respectively (Figure 6).

## Discussion

The rapid spread of COVID-19 has upended the entire health system globally with a profound negative effect on many essential health services, including routine immunization. Our study showed a slump of between 3.7% and 3.5% in the coverage of immediate post-natal antigens as confirmed by the pre- and post-index case coverages for BCG and HBV0 from 85.8% to 82.1% and 63.5% to 60.0% respectively. Similarly, we noticed that the peak coverage period for both antigens was August 2019 well before the onset of COVID-19 pandemic. While lockdowns and physical distancing have been blamed for the observed depression in immunization performance, local challenges like vaccine stock-out that normally creates access problem in developing countries are not unlikely contributors. Roxanne, in his study, identified the historic squeeze in the global transportation network as a major constraint which affected vaccine distribution worldwide [19]. Furthermore, the pattern in this study homogenized with reports from other countries like Pakistan [20] and the United Kingdom [21] which recorded similar drop in immunization coverage. Improvement in the coverage rates of these antigens will not only reduce the existing burden but will also prevent the outbreak of hepatitis and tuberculosis in the State. Going further, analysis of co-administered vaccines at 14 weeks revealed a sinusoidal wavelike pattern throughout the study period. Of particular concern was the overall reduction in the average coverage rates for Penta 3, OPV3, PCV3 and IPV in the period under review. These findings were in complete agreement with the previous studies conducted on the impact of COVID-19 on uptake of routine immunization [22-24].

The declining trend in the coverage of these antigens especially, benchmark antigens like Penta-3 portends a clear and present danger to the country´s recently achieved polio-free status. The average drop-out rates for pre-and post-index case periods were 5.0% and 4.7% respectively, it is instructive to know that there was a negligible reduction of 0.3% in the comparative analysis. In this regard, Oyo State government should put machinery in motion to ensure instant closure of the gaps created by COVID-19 pandemic and its response, no matter how small the gaps may be. For terminal or end-of-schedule vaccines administered at 9 months, the study found a significant decline during the COVID-19 pandemic using extant pre-COVID trend as the baseline. The lowest coverage (13.0%) for Yellow fever was recorded in August 2020 as against 67.0% recorded in August 2019. The implication is that within a year, there was a drop of 54.0% in the coverage rate and this was in total agreement with other studies on the uptake of routine immunization in the COVID-19 era [22,23]. However, there was an overall reduction of 15.9% coverage rate during the pandemic. This reduction may put the state at the risk of yellow fever outbreak bearing in mind its proximity to Kwara State where an outbreak was reported in 2017 [25]. For this reason, a strong epidemiologic watch is duly recommended. In the same vein, measles also suffered a loss of 12.4% in coverage rate during the study period (pre- and post-COVID-19). This loss may endanger the total national efforts at measles elimination. The observed depression in immunization coverage was not surprising given the inability to reach the target ceiling of 100.0% in the planned fixed and outreach immunization sessions, most especially during the post-index case period. The impact of non-pharmacologic measures as they relate to movement restriction was considerable in this regard.

## Conclusion

Decreased vaccination coverage for vaccine-preventable diseases could cause parallel outbreaks with COVID-19 and further exacerbate the strain on health systems struggling to end the acute phase of this pandemic. Therefore, as the dramatic second wave unfolds, the government of Nigeria must take deliberate steps to strike a balance between a fresh lockdown and the imperative of uninterrupted social service. In this wise, it must remain committed to a timely vaccination programme, most especially to build herd immunity and protect health care workers through provision of personal protective equipment and good disinfecting practices for vaccination clinics.

**Study limitation:** we used aggregated data that were not differentiated in socio-demographic characteristics, hence associations between variables were difficult to establish; therefore, inferences on causality may not be established.

**Interpretation:** the impact of COVID-19 pandemic has reduced the coverage rate of antigens used for routine immunization and the implication was that more children had already missed the live-saving vaccines. Hence, the risk of outbreak of vaccine-preventable diseases in the state is high.

**Generalizability:** the findings of the study could be generalized to the whole country. Looking at the proximal similarity model, the target population is the same throughout the country; the same data are collected on the same age group of people by the health workers (routine immunization focal persons) with similar training background. In addition, all the states of federation experienced lockdown at one time or the other.

### What is known about this topic

- *COVID-19 pandemic is known to cause economic and social problems;*
- *Immunization is one of the most successful and cost-effective public health interventions;*
- *Immunization reduces the outbreak of vaccine preventable diseases; routine immunization coverage depends on the client patronage to health facilities;vaccine preventable diseases are known to cause increased morbidity and mortality*.

### What this study adds

- *The immunization coverage for all the antigens was on the downward trend;*
- *Lockdown and other non-pharmacologic interventions reduced the coverage rate of routine immunization;*
- *Routine immunization sessions were disrupted during the pandemic; overall reduction in the coverage rates of yellow fever and HBV0 were15.9% and 19.2% respectively*.
